# Differential Immunological Responses of Adult Domestic and Bighorn Sheep to Inoculation with *Mycoplasma ovipneumoniae* Type Strain Y98

**DOI:** 10.3390/microorganisms12122658

**Published:** 2024-12-21

**Authors:** Sally A. Madsen-Bouterse, David R. Herndon, Paige C. Grossman, Alejandra A. Rivolta, Lindsay M. Fry, Brenda M. Murdoch, Lindsay M. W. Piel

**Affiliations:** 1 Department of Veterinary Microbiology and Pathology, Washington State University, Pullman, WA 99164, USA; sally.madsenbouterse@wwcc.edu (S.A.M.-B.); alejandra.rivolta@wsu.edu (A.A.R.); lfry@wsu.edu (L.M.F.); 2 USDA-ARS Animal Disease Research Unit, Pullman, WA 99164, USA; david.herndon@usda.gov (D.R.H.); paige.grossman@usda.gov (P.C.G.); 3 Department of Animal, Veterinary and Food Sciences, University of Idaho, Moscow, ID 83844, USA; bmurdoch@uidaho.edu

**Keywords:** *Mycoplasma ovipneumoniae*, bighorn sheep, domestic sheep, inoculation, cellular immune response, cytokine, antibody

## Abstract

Bighorn sheep (BHS) populations have been reported to experience high levels of morbidity and mortality following infection with *Mycoplasma ovipneumoniae*. This contrasts with the subclinical presentation in domestic sheep (DS). Understanding this difference requires baseline knowledge of pre- and post-infection immune responses of both species. The present study identifies differences in leukocyte phenotypes between adult BHS and DS before and after intranasal inoculation with 1 × 10^8^ *Mycoplasma ovipneumoniae*. Prior to inoculation, BHS were confirmed to have a higher abundance of leukocyte CD14 and serum concentrations of IL-36RA. In contrast, DS had a higher leukocyte abundance of CD16 in addition to previously observed integrin markers and CD172a, as well as greater serum TNF-α concentrations. Within 15 days of inoculation, BHS displayed signs of mild respiratory disease and *M. ovipneumoniae* DNA was detected on nasal swabs using a quantitative PCR; meanwhile, DS exhibited few to no clinical signs and had levels of *M. ovipneumoniae* DNA below the standard curve threshold. Immunologic markers remained relatively consistent pre- and post-inoculation in DS, while BHS demonstrated changes in the peripheral leukocyte expression of CD172a and CD14. Circulating serum IL-36RA decreased and CXCL10 increased within BHS. These findings highlight significant differences in cellular immunity between BHS and DS, raised and housed under similar conditions, prior to and following inoculation with *M. ovipneumoniae*.

## 1. Introduction

*Mycoplasma ovipneumoniae* is a bacterium reported to infect the respiratory tract of animals within the subfamilies Caprinae and Capreolinae [1]. *M. ovipneumoniae* has been reported to be the primary causative agent of a multifactorial polymicrobial pneumonia owed to its ability to compromise mucociliary clearance, thereby allowing secondary pathogens to colonize the lower respiratory tract [2,3,4]. Secondary pathogens in this disease complex can include *Mannheimia haemolytica*, *Bibersteinia trehalose*, and *Pasteurella multocida* [2,3,5,6]. The resulting disease state is considered chronic non-progressive pneumonia in domestic sheep (DS) and epizootic polymicrobial bacterial pneumonia in bighorn sheep (BHS) [2,5,7]. These alternate designations allude to the difference in morbidity, clinical signs, and mortality, death, observed between DS and BHS [8]. In DS, animals may develop a transient increase in respiration, mild coughing, and decreased weight gain, but overall, the disease favors a subclinical presentation [5,9,10]. Evidence supporting the subclinical nature in DS include lesions being found in the lungs of apparently healthy DS at the time of slaughter and farm-level prevalence being as high as 88% [6,11,12]. In contrast, BHS appear to be more susceptible to polymicrobial pneumonia, exhibiting a pattern of all-age die-offs and poor lamb recruitment for several years following an initial outbreak [2,3,7,13]. Additionally, recent work has demonstrated that some BHS that recover from *M. ovipneumoniae* infection continue to shed the bacterium [14,15,16], representing a source of infection and possibly re-infection for the remaining members of the herd.

Previous research has aided in determining potential reasons for differential susceptibility of BHS to respiratory disease. Peripheral blood analyses revealed that BHS have a higher number of circulating neutrophils compared to DS, whereas DS have a higher proportion of peripheral blood mononuclear cells (PBMCs) [17]. Further characterization of peripheral neutrophils demonstrated a higher proportion of CD14-positive cells with a higher marker density in BHS. This is in contrast to integrins, which tend to be expressed at higher levels in DS neutrophils [17,18]. It has yet to be determined if these differences are further impacted following inoculation with *M. ovipneumoniae*.

There are several previously published studies involving the inoculation or exposure of DS and/or BHS to *M. ovipneumoniae* [7,9,10]. The interpretation of those data is complicated by several factors, including a lack of inoculum quantification, the lack of any definition of the strain(s) present in the inoculum, and the possible presence of the secondary pathogens mentioned above. While this body of work has significantly advanced our understanding of the etiology of respiratory disease in BHS and DS, the characterization of the immune response to *M. ovipneumoniae* as a sole pathogen will allow for a more detailed understanding of pathogenesis. Therefore, the goal of this study was to compare the immune responses of naïve adult BHS and DS following monomicrobial inoculation with a single strain of *M. ovipneumoniae*.

## 2. Materials and Methods

### 2.1. Propagation of M. ovipneumoniae Inoculum

The type strain of *Mycoplasma ovipneumoniae* (NCTC 10151 [Y98]; American Type Culture Collection, ATCC^®^ 29419^TM^, Manassas, VA, USA) was first cultured under axenic conditions in liquid medium (Remel^TM^ SP4 Glucose Broth, Thermo Fisher Scientific, Waltham, MA, USA) and archived as glycerol stocks (15% glycerol and 10% phosphate buffered saline). Glycerol stocks were utilized to initiate the co-culture of *M. ovipneumoniae* with an ovine embryonic fibroblast cell line (A113), as previously described [19,20].

### 2.2. Animals, Inoculation, Monitoring, and Sampling

All animals were maintained under institutional animal care and use protocols approved by the Washington State University Institutional Animal Care and Use Committee (ASAF 4885, 30 August 2018). Five domestic sheep and five bighorn sheep were utilized for this study. Domestic sheep (DS, *Ovis aries*) included one Suffolk female (eight years old) and four Columbia–Suffolk cross females (six years old). Bighorn sheep (BHS, *Ovis canadensis*) (three males and two females) were eight years old at the time of the study. All sheep were born on site and maintained at a USDA animal research facility (Pullman, WA, USA). DS and BHS were separated from their ewe at birth and hand-raised. Colostrum was collected from each ewe and fed to their respective lambs within 24 h, after which the lambs were switched to lamb milk replacer until weaning. DS and BHS were housed by species and fed similar diets consisting of alfalfa hay, timothy hay, orchard hay, and alfalfa pellets. BHS were approximately double the weight of domestic sheep at inoculation. Animals displaying signs of respiratory disease prior to study inoculation underwent a full respiratory panel that included bluetongue virus, epizootic hemorrhagic disease, bovine respiratory syncytial virus, bovine viral diarrhea virus, infectious bovine rhinotracheitis, and parainfluenza-3 virus, all without detection. Additionally, all animals were periodically screened via nasal swabs to confirm they remained *M. ovipneumoniae*-free prior to inoculation.

Sheep were inoculated intranasally [9] with approximately 1 × 10^8^ bacteria [21] with volume split equally between the two nares. Following inoculation, animals were monitored daily for the development of clinical signs consistent with respiratory disease (e.g., coughing, nasal discharge, lethargy, etc.).

The nares were swabbed prior to inoculation and on days 5, 7, 11, 14, 18, 21, 25, and 28 post-inoculation. Briefly, nasal swabs (BD Universal Viral Transport Collection Kit; BD Biosciences, Franklin Lakes, NJ, USA) were inserted along the ventromedial aspect of the nasal cavity and gently twisted for approximately 5 s to avoid excessive irritation or trauma to the nasal cavity. A single swab was utilized for sample collection from both nares. Blood samples were collected for serum and leukocyte preparations via jugular venipuncture prior to inoculation and on days 1, 3, 5 (serum only), 7, 14, 21, and 28 post-inoculation. Blood for leukocyte preparations was collected into the ethylenediaminetetraacetic acid (EDTA) anticoagulant.

### 2.3. M. ovipneumoniae and Leukotoxin A PCR

DNA extractions from nasal swabs were performed using the QIAamp DNA Mini kit (Qiagen, Hilden, Germany) according to the manufacturer’s protocol for blood and bodily fluids, with a modified elution volume of 100 μL, and a qPCR was performed as previously described [22]. The absolute copy number of *M. ovipneumoniae* DNA was determined via standard curve cycle threshold (Cqs) values (10^6^ to 10^1^ copies). If detections were noted below the limit of quantification, they were given a value of 10 copies. Additionally, the detection of the *leukotoxin A* (*lktA*) gene was performed with DNA extracted from nasal swabs and following the PCR protocol described by Fisher et al., with stagnant growth requiring three colony-forming units for detection [23].

### 2.4. Flow Cytometric Assessment of Leukocyte Clusters of Differentiation Molecules

Peripheral blood leukocytes (PBLs) were prepared from 5 mL whole blood for flow cytometric analyses, as previously described [17]. Ethylenediaminetetraacetic acid (EDTA) vacutainer tubes were employed for blood collection. Collected blood was mixed with 0.5 mL of 1× PBS with 200 μg/L of EDTA (PBS-EDTA) in a 50 mL conical. Red blood cell lysis was completed by adding 36 mL of double distilled water, followed by 20 seconds of time with gentle inversion. Afterwards, 4 mL of 10× PBS with 2 mg/L EDTA was added to restore physiologic osmolarity. This suspension was centrifuged at 600× *g* for 10 min with the resultant supernatant discarded. The total leukocyte pellet was resuspended in 500 μL of PBS-EDTA and red blood cell lysis repeated with smaller volumes. Centrifugation was repeated with the pellet being resuspended in PBS-EDTA.

Immunostaining was performed with antibodies previously shown to bind leukocyte clusters of differentiation (CD) molecules on PBLs from both DS and BHS [17]. PBLs (2 × 10^5^ per well) were incubated on ice for 15 min with monoclonal primary antibodies listed in Supplementary Table S1. Plates were washed in flow cytometry first wash buffer (FWB) three times via centrifugation at 730× *g* for 3 min at 4 °C prior to the addition of fluorescent-conjugated secondary antibodies diluted 1:1000 in FWB. Plates were incubated on ice for 15 min protected from light, centrifuged as above, and washed two times in FWB without serum and finally resuspended in 1× PBS containing 1% formaldehyde.

Data acquisition was performed with a CytoFLEX S flow cytometer and CytExpert software (version 2.3; Beckman Coulter Life Sciences; Indianapolis, IN, USA). During acquisition, a forward scatter area and forward scatter height plot was utilized for single-cell selection. A combination of autofluorescence and side-scatter was utilized to gate neutrophils relative to peripheral blood mononuclear cells (PBMCs) and eosinophils [17], and acquisition was set to stop when 20,000 neutrophilic events were recorded. Leukocyte population proportions and median fluorescence intensity (MFI) were analyzed post-acquisition using FCS Express (version 6; De Novo Software; Pasadena, CA, USA). Controls included wells without primary antibodies which were used when setting gates to differentiate negative and positive populations during post-acquisition analyses.

### 2.5. Multiplex Assay of Serum Cytokines/Chemokines

Serum cytokine concentrations were assayed in duplicate using a commercially available multiplex immunoassay (Milliplex MAP Ovine Cytokine/Chemokine Magnetic Bead Panel 1, EMD Millipore Corp., Bedford, MA, USA) as per the manufacturer’s instructions. In short, sera were thawed at 4 °C, centrifuged at 10,000× *g* for 10 min to remove debris, and diluted 1:2 with sample diluent. MFI data were acquired with a Luminex LX200 (EMD Millipore Corp). Post-acquisition analyses were performed with Belysa^TM^ Immunoassay Curve Fitting Software (version 1.1.0; EMD Millipore Corp) using the 5-parameter logistic curve fitting model.

### 2.6. Immunocapillary Assay

Antibody responses were assessed via the immunocapillary assay (Abby, Protein simple, San Jose, CA, USA) with a 12–230 kDa separation module kit and detection module kit (ProteinSimple), according to the manufacturer’s instructions. Antigen was prepared from the type strain of *M. ovipneumoniae* harvested from A113 co-culture [19] by resuspending the bacterial pellet in 400 μL of phosphate buffered saline (PBS). Thereafter, the sample was frozen at −80 °C and thawed before antigen quantification using the Qubit™ Protein Assay Kit (Freeland Park, UK), in line with the manufacturer’s instructions. Prepared antigen was diluted to 0.2 mg/mL in a 0.1× sample buffer (ProteinSimple). Sera (primary antibodies) were applied at a 1:125 dilution in antibody diluent. The secondary antibody employed an anti-sheep IgG conjugated to HRP (cat# HAF016; R&D systems, Minneapolis, MN, USA) at a 1:50 dilution. Data were analyzed using Compass for SW software (version 6.2.0; ProteinSimple).

### 2.7. Statistical Analysis

Normal distribution was assessed based on species and marker using JMP version 17.2.0. Data with a normal distribution underwent a two-way analysis of variance (ANOVA) to determine differences due to the following variables: species, day, or the interaction of the previous two variables. Following two-way ANOVA, each pair-wise analysis was completed within the species using Student’s *t*-test to determine differences between days within species. Non-normal data employed general linearized mixed model to test for differences due to species, day, or the cross of species and day. Pairwise analysis for non-normal data used Wilcoxon/Kruskal–Wallis tests for each pair. Differences were considered significant at *p*-value < 0.05 for all the analyses.

## 3. Results

### 3.1. Post-Inoculation Detection of M. ovipneumoniae DNA and Measured Clinical Signs Consistent with Upper Respiratory Disease

**Figure 1:**
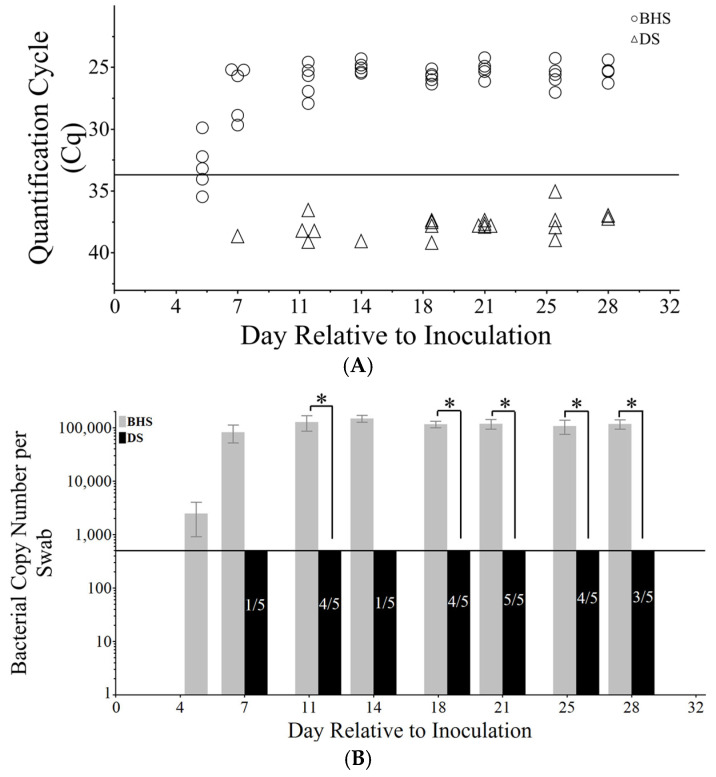
*M. ovipneumoniae* detected on nasal swabs using a quantitative PCR in DS and BHS. (**A**) Cycle thresholds (Cq) measured from DNA extracted from nasal swabs. BHS are represented by open circles (○) and DS are represented by open triangles (Δ). The solid line is the standard curve limit of quantification (33.52 Cq) associated with ten copies of genomic equivalents. (**B**) Bacterial genomic equivalents calculated on a per-nasal-swab basis. The line indicates the limit of quantification (500 copies per swab) at the standard which measures ten bacterial copies. Fractions in DS bars indicate the number of subjects detected per the five animals enrolled in the study. Asterisks indicate a *p*-value < 0.05 and error bars represent the standard error.

It is generally accepted that *M. ovipneumoniae* predisposes carriers to other pneumonia causing bacteria that could express the cytotoxin *leukotoxin A*, resulting in severe pleuropneumonia in the case of BHS [2,3,24]. A PCR was used to test for the presence of *leukotoxin A* (*lktA*) from nasal swabs for all study animals (Supplementary Figure S1). All BHS and DS were found to be negative for *lktA*. Prior to inoculation, *M. ovipneumoniae* was not detected on nasal swabs isolated from either sheep species (Supplementary Figure S2, [25]). Following inoculation, *M. ovipneumoniae* DNA was found to be above the limit of quantification of the quantitative PCR (qPCR) within nasal swabs sampled from BHS (Figure 1). Quantities began to increase five days post-inoculation and peaked around day 14 at approximately 100,000 DNA copies per nasal swab (Figure 1B). In contrast, the *M. ovipneumoniae* copy number was outside of the range of the standard curve for DS at all sampling times, where post-inoculation day 5 resulted in zero detections within DS (Figure 1A,B). The first detection within DS was day 7 post-inoculation from DS 13-2, and week 21 post-inoculation was the only timepoint where all five DS had detections of *M. ovipneumoniae* DNA (Figure 1B).

**Figure 2:**
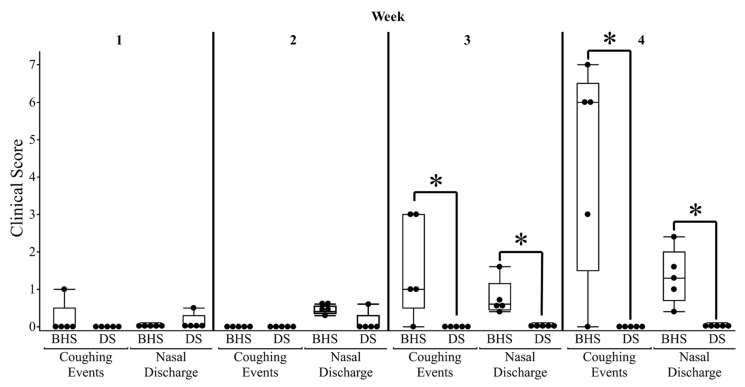
Clinical signs noted in bighorn and domestic sheep following inoculation with *M. ovipneumoniae*. Coughing was scored daily based on presence (1) or absence (0). The weekly clinical score was calculated by summing the daily scores for the week. In contrast, nasal discharge severity was observed twice a day and marked as a 0 to 5, where 0 = no discharge, 1 = mild discharge, 2 = moderate discharge, 3 = moderately severe discharge, 4 = severe discharge, and 5 = excessive discharge. Each animal’s weekly nasal discharge score was calculated by averaging the week’s scores and dividing by the number of times the animal was observed. Each point represents an individual animal enrolled in the study and asterisks indicate a *p*-value < 0.05.

Regarding the observation of clinical signs, BHS 11-B, 11-E, and 11-G were observed to have nasal discharge and periodic coughing starting at 15 days post-inoculation (Figure 2). One DS, DS 11-5, had transient nasal discharge in the first and second week of post-inoculation that resolved by week three. There were no coughing events observed in the DS species.

### 3.2. Peripheral Leukocyte Profile Pre- and Post-Inoculation

**Figure 3:**
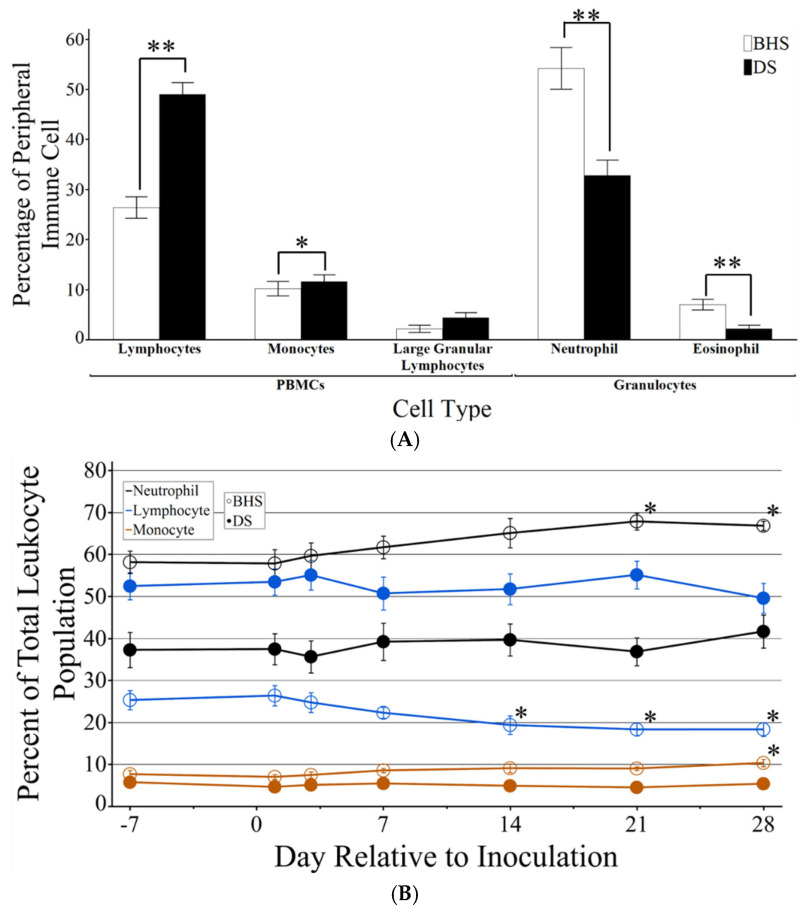
Peripheral leukocyte profiles in BHS and DS. (**A**) Percent of resting cellular populations calculated by observing 100 cells during manual differentials. Open bars are BHS and closed bars are DS. (**B**) Mean percent of neutrophils (black), lymphocytes (blue), and monocytes (orange) from the total leukocyte population for each species over the course of observation using flow cytometry. Bighorn sheep are represented by open circles (○) and domestic sheep are represented by solid circles (●). Both graphs have standard error incorporated and a *p*-value < 0.05 indicated by an * or <0.005 indicated by **. Significance in (**B**) is a comparison between the labeled day and day minus seven.

**Figure 4:**
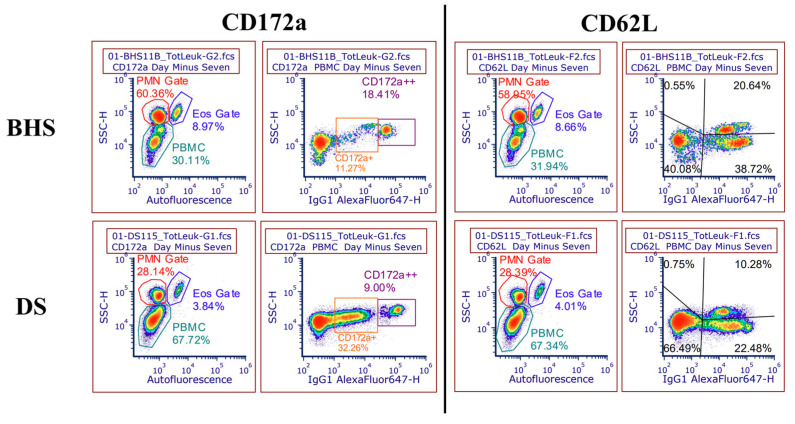
Gating strategies for leukocyte cell type and adhesin markers. Each density dot plot is from seven days prior to inoculation and is based on the total leukocyte population. Side-scatter (SSC) and autofluorescence were used to differentiate between neutrophils, eosinophils, and peripheral blood mononuclear cells (PBMCs) in the left-hand image for each cluster of differentiation. The right-hand image for either marker shows the gating used to distinguish dim and bright populations of CD172a, as well as upper right and lower right populations of CD62L-positive lymphocytes.

Similar to previous work [17], manual differential cell counts in blood from seven days prior to inoculation demonstrated that BHS have higher numbers of circulating neutrophils and lower numbers of circulating PBMCs as compared to DS. The present work expanded on prior results by demonstrating that lymphocytes are predominantly responsible for the larger numbers of baseline PBMCs in DS (Figure 3A). In addition to the assessment of baseline differential leukocyte counts in adult BHS and DS, cell populations were followed over the course of inoculation using both manual differential counts and flow cytometry (Supplementary Figure S3). The traditional microscopic assessment of cell types (manual differential counts) and flow cytometry demonstrated a high level of agreement for cell population profiles (Supplementary Figure S3). Flow cytometry was employed for the remainder of the study, as this technique examines approximately 200-fold more cells per sample and reduces bias that can be associated with manual differentials. Figure 3B illustrates the peripheral leukocyte profile over the course of inoculation using the pan-leukocyte marker CD18 in conjunction with side-scatter and autofluorescence, allowing for differentiation between neutrophils, monocytes, and lymphocytes. Examples of this gating strategy are depicted in Figure 4. While the inoculation of DS did not result in significant changes in cell type distribution, the inoculation of BHS resulted in an increase in neutrophils (approximately 9%) and monocytes (approximately 3%), along with a decrease in lymphocytes (approximately 7%) (Figure 3B).

### 3.3. Immune Cell Surface Markers

Surface markers that were validated in both species were used for flow cytometry (Supplementary Table S1) [17]. Beyond integrin CD18, there were two other integrin markers (CD11a and CD11b), adhesins (CD172a and CD62L), and immunologic function (CD14 and CD16) markers employed. The median fluorescence intensity (MFI) of markers prior to inoculation were comparable with previous data comparing DS and BHS peripheral leukocytes (Supplementary Figure S4) [17]. Adhesin markers were subdivided into subpopulations, where CD172a consisted of a dim (+) and bright (++) population, while CD62L exhibited two levels of complexity, denoted as upper right (UR) and lower right (LR) (Figure 4). Based on side-scatter characteristics [17,26], it is presumed that the upper right population of CD62L-positive PBMCs represents monocytes, while the lower right population represents lymphocytes.

### 3.4. Time Course Changes in Surface Integrin Markers

**Figure 5:**
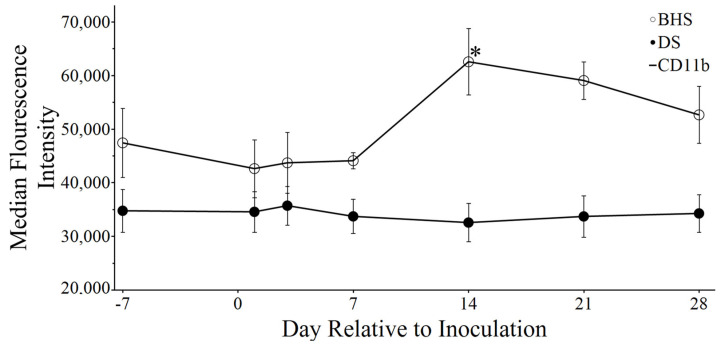
CD11b MFI in PBMCs following inoculation with *M. ovipneumoniae*. The asterisk indicates a *p*-value < 0.05 and is comparing the day marked to day minus seven.

The pan-leukocyte marker CD18 pairs with either CD11a, CD11b, or CD11c to generate a heterodimeric surface complex known as an integrin, where CD18 is the beta integrin and CD11 -a, -b, and -c are the alpha integrins L, M, and X, respectively [27]. BHS neutrophils did not exhibit significant changes in integrin abundance over the inoculation time course (Supplementary Table S2). In contrast, BHS PBMCs demonstrated a significant increase in the MFI of CD11b-positive cells (Figure 5).

### 3.5. Time Course Changes in Surface Adhesins

**Figure 6:**
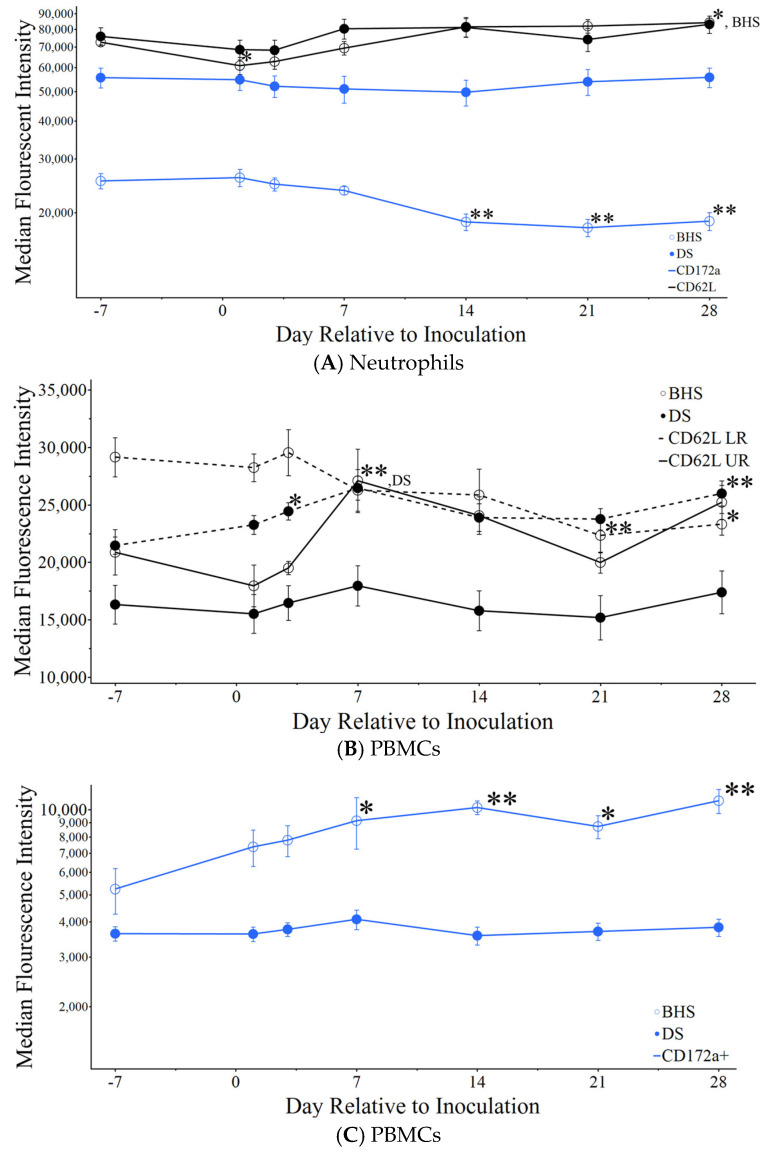
Adhesin expression changes in neutrophils and PBMCs following inoculation. CD172a is blue and CD62L is black, with UR having solid lines and LR having dashed lines. (**A**) The median fluorescence intensity for either marker in neutrophils, (**B**) the MFI for CD62L UR and LR measured in PBMCs, and (**C**) the MFI for CD172a+ in PBMCs. Significance is marked to the upper right of the graphed data point, where points that overlap are indicated by either DS or BHS. A *p*-value < 0.05 is indicated by * and <0.005 indicated by **, where the labeled day is compared to day minus seven.

CD172a can be responsible for the migration of cells into alveoli [28], while CD62L is responsible for lymphoid tissue homing [29]. The MFI of these markers on BHS neutrophils demonstrated an increase in CD62L that peaked at day 14 post-inoculation, with a contrasting decrease in CD172a beginning at day 14 post-inoculation (Figure 6A). The BHS monocyte expression of CD62L (CD62L UR) increased between days one and seven post-inoculation, while expression in BHS lymphocytes (CD62L LR) decreased over the course of the study. In contrast, CD62L in lymphocytes from DS increased in the first seven days post-inoculation and then plateaued (Figure 6B). Unlike BHS neutrophils, BHS PBMCs increased in abundance of CD172a+ immediately post-inoculation, a trend that continued throughout the duration of the study (Figure 6C).

### 3.6. Time Course Changes in CD14 and CD16

**Figure 7:**
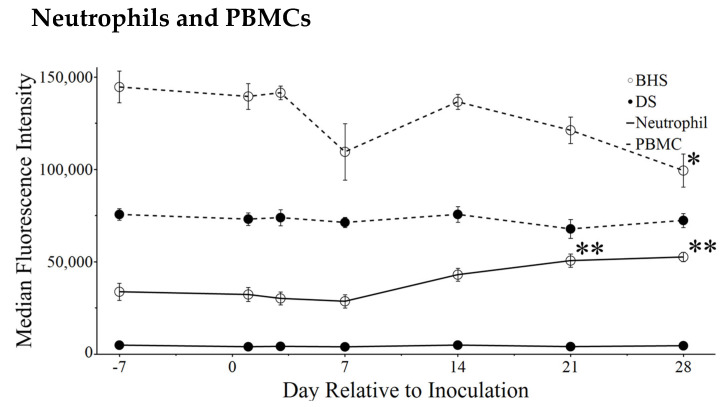
CD14 abundance in neutrophil and PBMC populations following inoculation. Solid lines are neutrophils and dashed lines are PBMCs. Asterisks mark the day in question for comparison to day minus seven, where * is a *p*-value < 0.05 and ** is a *p*-value < 0.005.

CD14 is known for its role in Toll-like receptor 4 (TLR4) stimulation following the cellular recognition of lipopolysaccharide (LPS) [30]. In BHS, the MFI of CD14 on neutrophils increased significantly by day 21 post-inoculation and was higher than that of DS neutrophils for the entire time course. CD14 abundance was higher in PBMCs compared to neutrophils regardless of species. Interestingly, BHS CD14 MFI decreased in PBMCs but remained consistent in DS over the course of the study (Figure 7). CD16 is a well-known Fcγ receptor that mediates antibody-dependent cellular cytotoxicity [31]. Like CD14, the MFI of CD16 increased on BHS neutrophils following inoculation, but this increase was moderate and did not reach statistical significance (Supplementary Figure S5). Data associated with the percent of the cellular population can be found in Supplementary Table S3. Notably, the percentage of gated neutrophils which were positive for each marker tested remained within 98–100%. In contrast, the percentage of gated PBMCs followed similar trends as those seen during MFI analyses with markers CD11b, CD62L UR, CD62L LR, and CD172a+. The only PBMC marker tested which showed an increase in the gated population with a decrease in MFI was CD14. CD16 showed a similar increase in the gated population of PBMCs; however, the MFI did not change over the time course.

### 3.7. Measurement of Serum Cytokines from Baseline and Post-Inoculation

**Figure 8:**
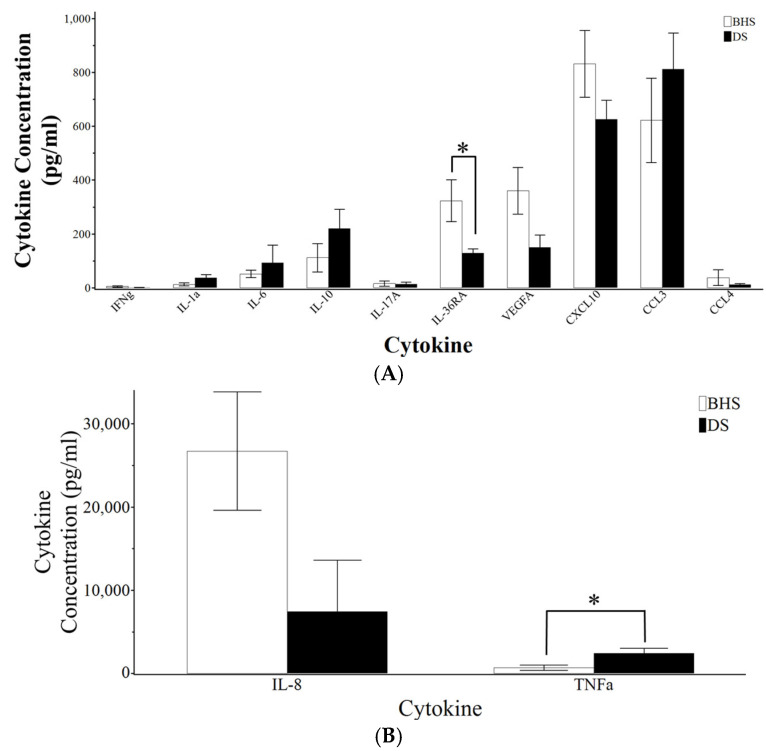
Pre-inoculation serum cytokine concentrations. (**A**) Cytokines with values below 1000 pg/mL and (**B**) those with values above 1000 pg/mL. A *p*-value < 0.05 is marked with an asterisk.

**Figure 9:**
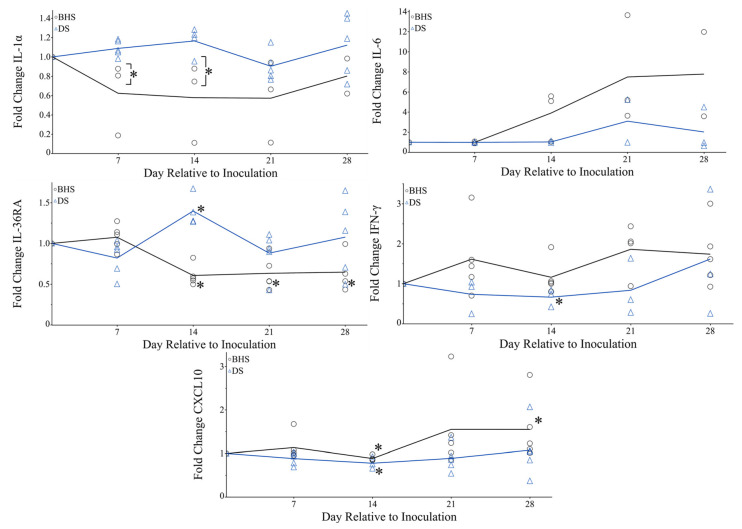
Serum cytokine fold change relative to day one post-inoculation. Serum concentrations measured each week were divided by day one cytokine concentrations to achieve a relative fold change. BHS are represented by open black circles, while DS are represented by open blue triangles. A *p*-value < 0.05 is labeled with an asterisk and represents significant difference to day one or between species.

Sera from DS and BHS were assessed for their variations in IFN- γ, IL-1α, IL-1β, IL-4, IL-6, IL-8, IL-10, IL-17A, MIP-1α, MIP-1β, CXCL10, IL-36RA, TNF-α, and VEGFA using a bead-based multiplex immunoassay. The detection of IL-1β and IL-4 was limited, where only three samples were above the lower limit of quantification (LLoQ). All other cytokines and chemokines demonstrated quantitative measurements for the length of the study (Supplementary Figure S6). Pre-inoculation measurements allowed for the comparison between BHS and DS cytokines and chemokines on a species level (Figure 8). Serum TNF-α and IL-1α were significantly higher in DS. Alternatively, BHS species had higher serological levels of IL-36RA and VEGFA (*p*-value 0.0636). While species-based differences were not observed for the remainder of the cytokines measured, our data suggest that certain cytokine/chemokines levels were dissimilar between sheep species. Due to the inherent differences in cytokine quantities between species, the fold changes for IL-1α, IL-6, IL-36RA, IFN-γ, and CXCL10 were calculated weekly relative to day one post-inoculation (Figure 9). The fold change in IL-1α was significantly different between BHS and DS at days 7 and 14. BHS species demonstrated a near-significant increase in serum IL-6 when comparing day 1 post-inoculation values to either day 14 or 21 (*p*-value of 0.0636). The measurement of IL-36RA revealed that both species underwent significant concentration changes post-inoculation. Specifically, BHS had a decreased serum concentration of IL-36RA beginning at day 14, while DS had increased the concentration on day 14. DS returned to pre-inoculation IL-36RA levels on day 21.

Serum concentrations of IFN-γ began increasing in BHS at day seven post-inoculation. While DS were found to have increased levels of IFN-γ at day 28 post-inoculation, the result is solely due to animal DS 13-1 and does not represent a change within all study animals. Although IFN-γ did not reach a significant fold change within the BHS, CXCL10 serum concentrations resulted in a significant fold change at day 28 post-inoculation.

### 3.8. Antibody Response to M. ovipneumoniae Following Inoculation

**Figure 10:**
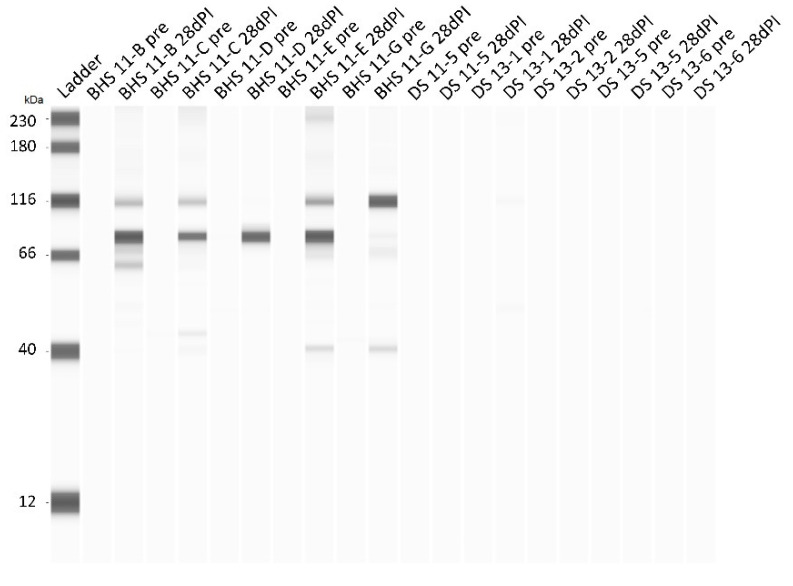
BHS and DS serum antibodies to Mycoplasma ovipneumoniae pre-inoculation and 28-days post-inoculation. The ladder and associated kDa values are in the first sample lane. Thereafter, the pre-sample is in the first lane for each animal in the inoculation study and is followed by the 28-day post-inoculation (dPI) serum sample.

The antibody response to *M. ovipneumoniae* was measured pre-inoculation and 28 days post-inoculation for all animals (Figure 10). While there were only minor detectable differences between reactivity pre-inoculation and 28 days post-inoculation within DS, there was a substantial increase in the immunoreactivity of BHS serum with *M. ovipneumoniae* antigens. A more detailed analysis of the BHS antibody response reveals that antibodies were being produced as early as day 14 post-inoculation (Supplementary Figure S7). The protein which was recognized by all BHS was estimated to be 83 kDa in size. Additionally, a protein of 40 kDa reacted with serum from BHS 11-B, 11-E, and 11-G, while a protein estimated to be 115 kDa was recognized by serum from BHS 11-B, 11-C, 11-E, and 11-G.

## 4. Discussion

The present study assessed the outcome of inoculating adult, hand-raised, bighorn, and domestic sheep with the bacterium *Mycoplasma ovipneumoniae*. Following inoculation, *M. ovipneumoniae* was detected in both BHS and DS species; however, detections in DS were largely transient and below the lowest standard via qPCR. This aligns with clinical observations being almost exclusively observed in BHS. BHS developed nasal discharge, coughing, and mild lethargy; however, they did not display signs consistent with severe respiratory distress. Notably, this study employed aged BHS and DS, which could either be mature enough to allow for an immunocompetent response or to cause immunocompromise due to aging. Within the field, it is generally accepted that BHS are more susceptible to disease than DS following *M. ovipneumoniae* exposure [2,7], and while our data are consistent with this premise, it suggests that there is reduced disease severity during monomicrobial infection. The removal of animals at birth and controlled environmental conditions presumes that other microbial respiratory flora associated with DS/BHS polymicrobial disease is absent. Support of this theory comes from testing for the presence of the *leukotoxin A* gene, a cytolytic toxin commonly carried by *Pasteurellaceae* species during polymicrobial pneumonia or chronic non-progressive pneumonia (Supplementary Figure S1) [2,32].

Data from this study are concordant with previous work (16) that demonstrates a species-based difference between blood profiles for DS and BHS (Figure 3A). Additionally, our data suggest that DS and BHS tend to rest in opposing inflammatory states, with DS trending toward pro-inflammatory homeostasis and BHS towards an anti-inflammatory homeostasis. The pre-inoculation median fluorescence intensity of CD markers (Supplementary Figure S4) and serum cytokine levels (Figure 8) demonstrates the higher abundance of integrins and CD172a in DS, which could allow for the faster tissue localization of inflammatory cells following pathogen exposure [27,28]. Similarly, TNFα and IL-1α represent early response cytokines that upregulate during an inflammatory reaction [33,34,35]. In contrast, BHS had higher levels of IL-36RA and VEGFA, which have been shown to skew the immune response to a more anti-inflammatory and healing state [36,37].

In addition to the pre-inoculation species differences, we observed immunologic changes following the *M. ovipneumoniae* inoculation of BHS. Alterations in CD62L, as shown in Figure 6, likely represent antigen-presenting cells, like monocytes, beginning to attach to lymphoid tissue (CD62L upper right) following the increased density of CD62L. Similarly, the resultant activation of lymphocytes (CD62L lower right) would likely decrease CD62L MFI, as seen in Figure 6 [29,33]. CD172a+ data suggest that PBMCs could mobilize to the lungs, while neutrophil abundance suggests a decreasing propensity for this anatomic locale (Figure 6). While suppurative (neutrophilic) inflammation is commonly diagnosed during epizootic pneumonia in BHS [13,38], it is feasible that the presence of *Pasterellaceae* is required to drive the neutrophilic response in the lung via the secretion of leukotoxin A [24,39]. The staining strategy within this study did not allow for dual labeling, and while additional reagents may be evaluated in the future to improve the characterization of DS and BHS leukocytes using flow cytometry, CD172a+ and CD11b abundance changes within PBMCs could not be associated with a specific cellular population. Dual staining with CD172a+ or CD11b and CD14 would aptly label the monocytic population to assess if the median fluorescence intensity increase was occurring within monocytes [40].

In addition to altered leukocyte cell surface markers, transitions with serum cytokines indicate immune system activation within BHS (Figure 8 and Figure 9). IL-6 values exhibit an increased in fold change (*p*-value of 0.0636) at days 14 and 21 in comparison to day 1 within BHS. IL-6 has been shown to promote the differentiation of B-cells to plasma cells through its effects on naïve and memory T-cells [41]. Therefore, one possible outcome of increased IL-6 could be antibody production, which also started occurring on day 14 post-inoculation (Figure 9 and Figure S7). Two other cytokine alterations that suggest immune system activation in BHS are IL-36RA and CXCL10. IL-36RA antagonizes the effects of other IL-36 cytokines which play a role in neutrophil chemotaxis, T-cell stimulation, and macrophage autophagy [36]. While IL-36RA levels started to decrease 14 days post-inoculation in BHS, it took until day 21 to see an increase in CXCL10. CXCL10 is otherwise known as interferon gamma-inducible protein (IP-10), which acts in a positive feedback loop to positively regulate the adaptive immune system [42]. Recent advances in CXCL10 research suggest the use of this cytokine as a biosensor for the severity of disease [42,43]. Notably, this cytokine can result in tissue damage if expression goes unchecked or does not become down-regulated [42].

Pre-inoculation CD14 abundance was higher within BHS species, and where inoculation with *M. ovipneumoniae* further increases neutrophilic surface abundance of CD14, it lowers CD14 surface abundance on PBMCs. This marker is responsible for signaling following exposure to bacterial lipopolysaccharide (LPS) [44,45], and while *M. ovipneumoniae* is not known to express LPS, *Pasteurellaceae* species present surface LPS [45,46,47,48]. This raises the question of what role CD14 plays during polymicrobial epizootic pneumonia within bighorn sheep, where *Mycoplasma ovipneumoniae* exposure may cause LPS sensitization, resulting in a severe inflammatory reaction when *Pasteurellaceae* species translocate into the lower respiratory tract. A similar concept has been theorized during bovine respiratory disease and is considered the synergy of infection between bovine herpes virus-1 (BHV-1) and *Mannheimia haemolytica*. Specifically, the increased messenger RNA expression of CD14 and toll-like receptor 4 following BHV-1 infection was correlated to higher mortality when cattle were exposed to *M. haemolytica* [49].

The present work suggests that DS PBMCs, serum cytokines, and immunoglobulins remain largely unaffected following *M. ovipneumoniae* exposure with the Y98 type strain at the present intranasal dose. Notably, the nasal swab testing of DS resulted in bacterial detection that was transient, infrequent, and below the lowest standard using a qPCR. This is consistent with recent work by Robinson et al., which determined the inconsistent shedding of *M. ovipneumoniae* DNA with absent clinical signs when inoculating DS with 3.5 × 10^8^ colony-forming units [21]. Future work should attempt to ascertain if there is a localized immune response that is immeasurable within peripheral blood or if there is a peripheral response when alternate strains, doses, or multi-strain infections of *M. ovipneumoniae* are encountered.

## 5. Conclusions

Based on these results, BHS and DS exhibit differing immune resting states, where BHS maintain a more anti-inflammatory state at homeostasis. These baseline differences may partially explain why BHS are more susceptible to pneumonia following exposure to *Mycoplasma ovipneumoniae*. The present work focuses on monomicrobial inoculation with *M. ovipneumoniae*, which resulted in only mild respiratory signs in BHS and had little effect on DS.

## Data Availability

The original contributions presented in the study are included in the article/Supplementary Materials; further inquiries can be directed to the corresponding author.

## Supplementary Materials

The following supporting information can be downloaded at: https://www.mdpi.com/article/10.3390/microorganisms12122658/s1. Table S1: Flow cytometry antibodies and their recognized markers; Figure S1: *Leukotoxin A-*carrying bacterial species were not detected in study animals; Figure S2: LM40 PCR on the detection of *M. ovipneumoniae* isolated from nasal swabs 26 days pre-inoculation; Figure S3: Manual and flow cellular percentages follow similar trends; Figure S4: Day minus seven species differences in the median fluorescence intensity of different clusters of differentiation; Table S2: Integrin marker values for neutrophils over the course of inoculation; Figure S5: The neutrophil MFI of CD16 over the course of inoculation; Table S3: Percent population changes for tested markers within PBMCs and neutrophils; Figure S6: Serum cytokines measured by multi-plex ELISA; Figure S7: Bighorn sheep serum antibody response to *Mycoplasma ovipneumoniae* over time.
