# Supplementary material for: Differential Immunological Responses of Adult Domestic and Bighorn Sheep to Inoculation with Mycoplasma ovipneumoniae Type Strain Y98
Source: Microorganisms. 2024 Dec 21;12(12):2658. doi: 10.3390/microorganisms12122658 (PMC11728652; doi:10.3390/microorganisms12122658)
Supplement: Supplementary file 1 [file microorganisms-12-02658-s001.zip › Supplemental Figure S1 LktA.pdf]

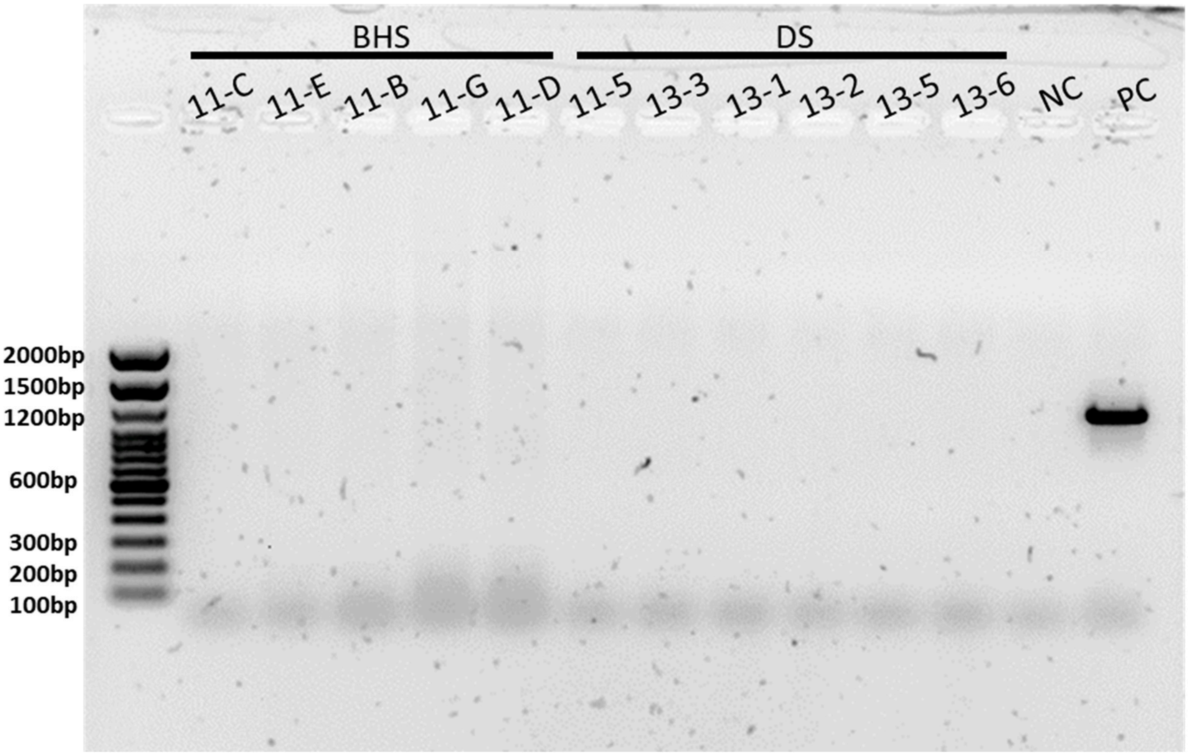

Supplemental Figure S1: *Leukotoxin A* carrying bacterial species were not detected in study animals. DNA extracted from nasal swabs underwent polymerase chain reaction assessing for *leukotoxin A* (*lktA*). BHS are the bighorn sheep and DS are the domestic sheep enrolled in the study. NC is the negative control and PC is the positive control. The expected band size is 1,145 base pairs.
