# Supplementary material for: Differential Immunological Responses of Adult Domestic and Bighorn Sheep to Inoculation with Mycoplasma ovipneumoniae Type Strain Y98
Source: Microorganisms. 2024 Dec 21;12(12):2658. doi: 10.3390/microorganisms12122658 (PMC11728652; doi:10.3390/microorganisms12122658)
Supplement: Supplementary file 1 [file microorganisms-12-02658-s001.zip › Supplemental Figure S2 LM40 gel.pdf]

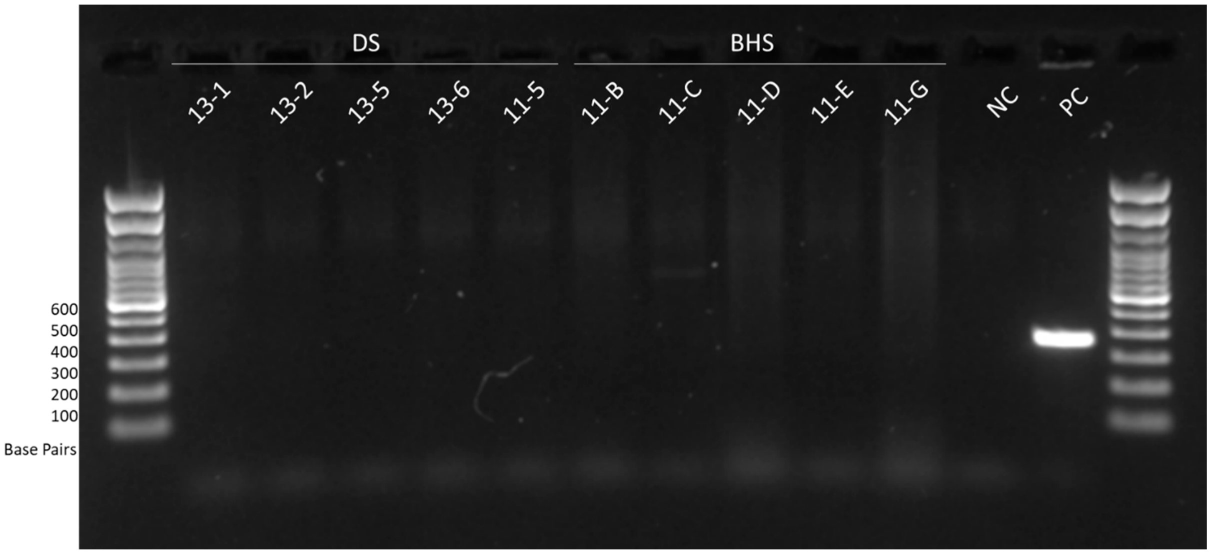

Supplemental Figure S2: LM40 PCR detection of *M. ovipneumoniae* isolated from nasal swabs 26 days pre-inoculation. LM40 PCR was run as indicated in McAuliffe et al [1].Domestic sheep are labeled with DS and their corresponding number while bighorn sheep are labeled with BHS and the corresponding animal number. NC is the negative control and PC is the positive control. The PC should run at 361 base pairs. The first ladder is labeled to depict the appropriate base pair size.

1. McAuliffe, L., et al., *Detection of Mycoplasma ovipneumoniae in Pasteurella-vaccinated sheep flocks with respiratory disease in England.* Vet Rec, 2003. **153**(22): p. 687-8.
