# Supplementary material for: Differential Immunological Responses of Adult Domestic and Bighorn Sheep to Inoculation with Mycoplasma ovipneumoniae Type Strain Y98
Source: Microorganisms. 2024 Dec 21;12(12):2658. doi: 10.3390/microorganisms12122658 (PMC11728652; doi:10.3390/microorganisms12122658)
Supplement: Supplementary file 1 [file microorganisms-12-02658-s001.zip › Supplemental Figure S3 Manual vs Flow.pdf]

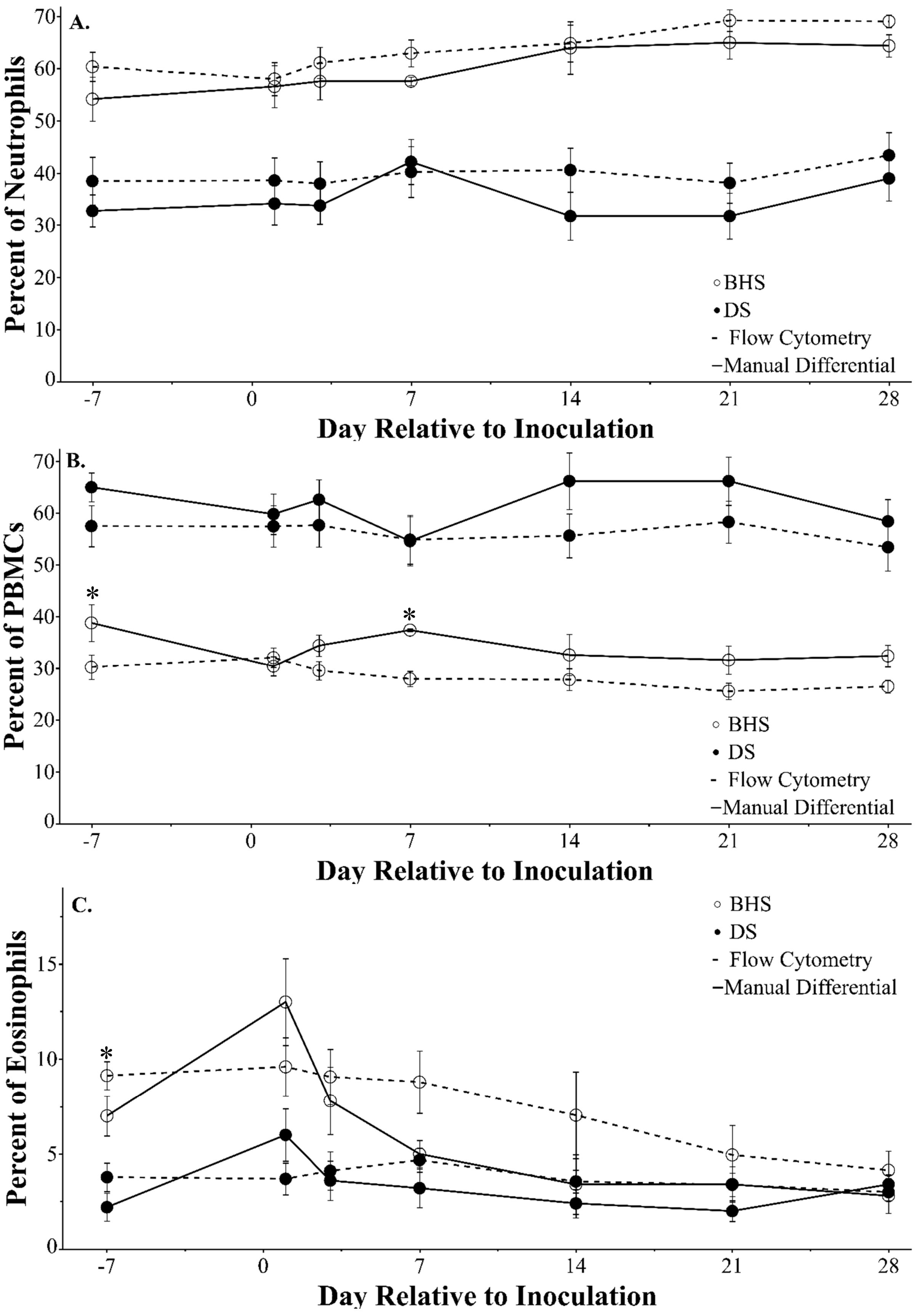

A.
Percent of Neutrophils
70
60
50
40
30
20
10
0
BHS
DS
Flow Cytometry
Manual Differential
-7
0
7
14
21
28
Day Relative to Inoculation
B.
Percent of PBMCs
*
*
BHS
DS
Flow Cytometry
Manual Differential
Day Relative to Inoculation
C.
Percent of Eosinophils
15
10
5
0
*
BHS
DS
Flow Cytometry
Manual Differential
Day Relative to Inoculation

Supplemental Figure S3: Manual and flow cellular percentages follow similar trends. (**A**) Neutrophils, (**B**) PBMCs, and (**C**) Eosinophil cellular population percentages over inoculation time course. Dashed lines are flow cytometry results and solid lines are manual differential counts. Open circles are BHS and closed circles are DS. Asterisks indicate a significant difference with p-value <0.05 between modalities employed.
