# Supplementary material for: Differential Immunological Responses of Adult Domestic and Bighorn Sheep to Inoculation with Mycoplasma ovipneumoniae Type Strain Y98
Source: Microorganisms. 2024 Dec 21;12(12):2658. doi: 10.3390/microorganisms12122658 (PMC11728652; doi:10.3390/microorganisms12122658)
Supplement: Supplementary file 1 [file microorganisms-12-02658-s001.zip › Supplemental Figure S4 CD Day Minus Seven.pdf]

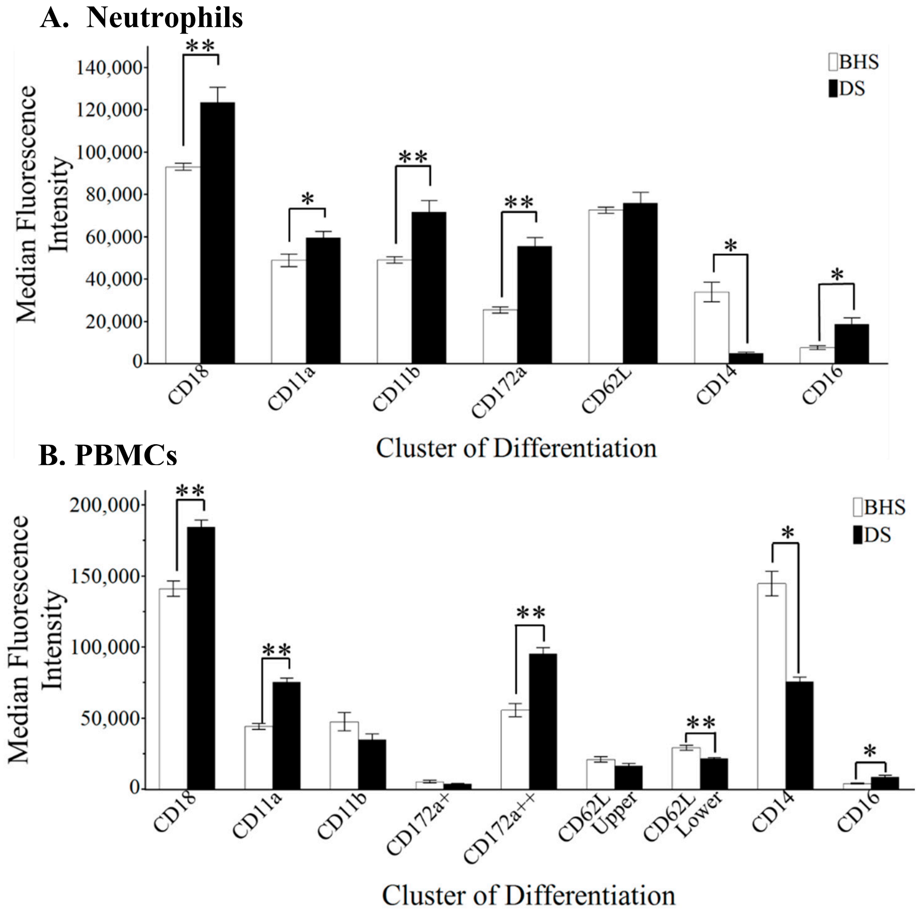

Figure S4: Day minus seven species differences in the median fluorescence intensity of different clusters of differentiation. Open bars depict bighorn sheep and closed bars domestic sheep. Mean fluorescence intensity of different cluster of differentiation (CD) molecules was assessed on isolated peripheral blood neutrophils (**A**) and peripheral blood mononuclear cells (**B**). The plus sign (+) denotes a dim population and the double plus sign (++) is a bright cellular population. Peripheral blood mononuclear cells had an upper and lower positive population of CD62L marked cells. Both graphs have standard error incorporated with * indicating a p-value of <0.05 and ** indicating a p-value <0.005.
