# Supplementary material for: Differential Immunological Responses of Adult Domestic and Bighorn Sheep to Inoculation with Mycoplasma ovipneumoniae Type Strain Y98
Source: Microorganisms. 2024 Dec 21;12(12):2658. doi: 10.3390/microorganisms12122658 (PMC11728652; doi:10.3390/microorganisms12122658)
Supplement: Supplementary file 1 [file microorganisms-12-02658-s001.zip › Supplemental Figure S5 CD16 MFI Neut.pdf]

Neutrophils

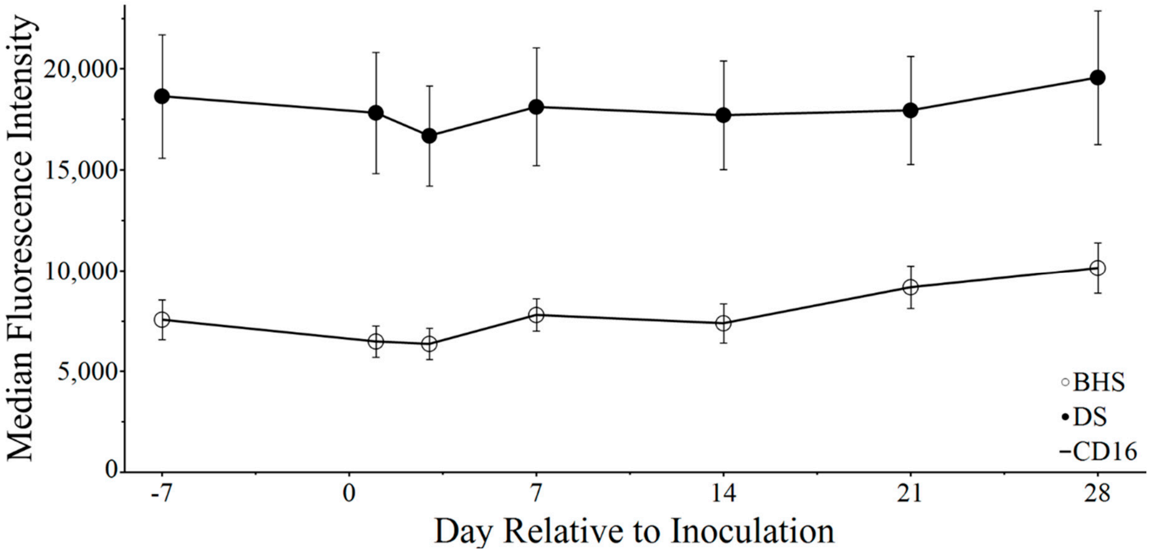

Supplemental Figure S6. Neutrophil MFI of CD16 over the course of inoculation. BHS are open circles and DS are closed circles. Error bars represent the standard error.
