# Supplementary material for: Differential Immunological Responses of Adult Domestic and Bighorn Sheep to Inoculation with Mycoplasma ovipneumoniae Type Strain Y98
Source: Microorganisms. 2024 Dec 21;12(12):2658. doi: 10.3390/microorganisms12122658 (PMC11728652; doi:10.3390/microorganisms12122658)
Supplement: Supplementary file 1 [file microorganisms-12-02658-s001.zip › Supplemental Figure S6 Serum Cytokines.pdf]

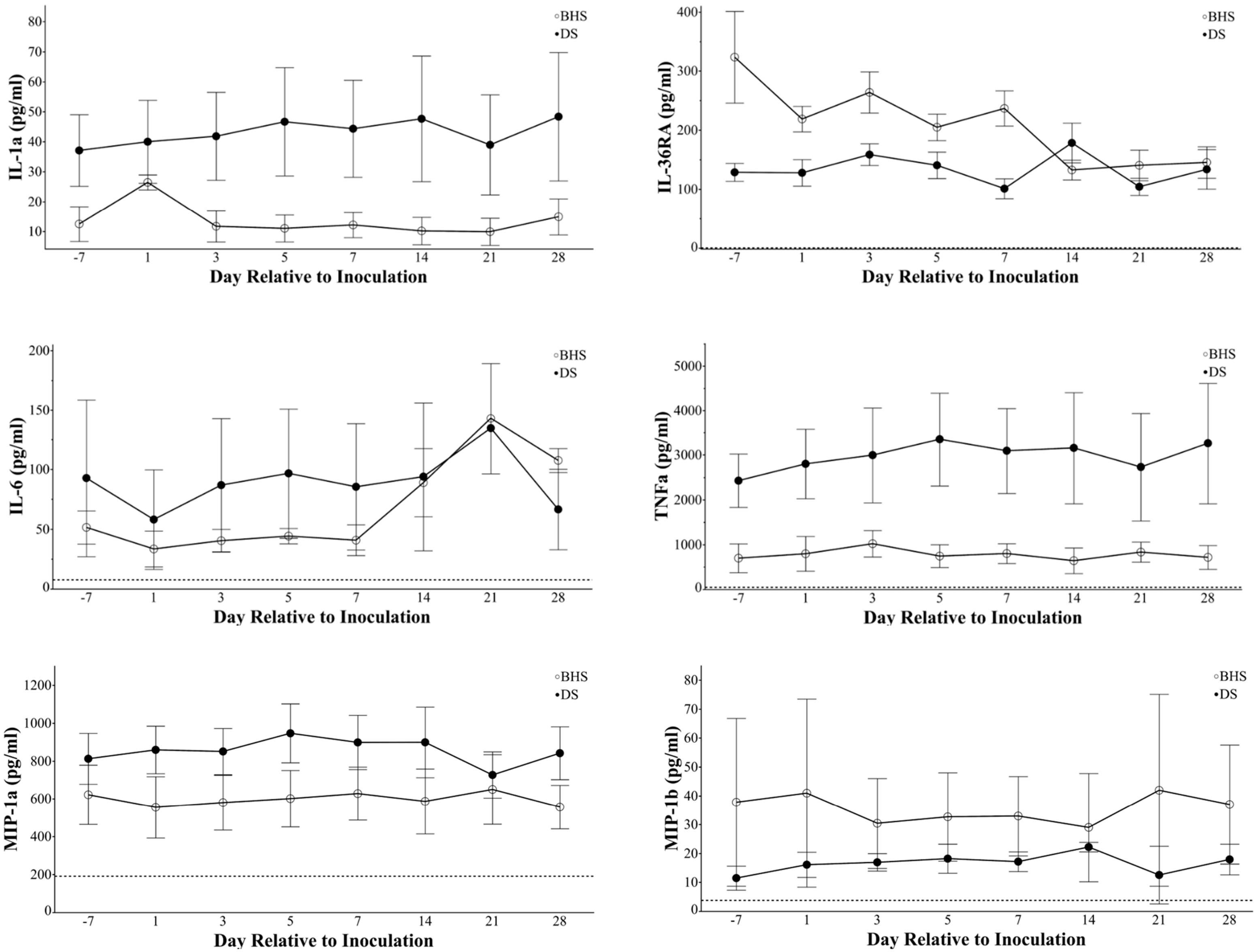
IL-1a (pg/ml)
○BHS
•DS
Day Relative to Inoculation
IL-36RA (pg/ml)
○BHS
•DS
Day Relative to Inoculation
IL-6 (pg/ml)
○BHS
•DS
Day Relative to Inoculation
TNFa (pg/ml)
○BHS
•DS
Day Relative to Inoculation
MIP-1a (pg/ml)
○BHS
•DS
Day Relative to Inoculation
MIP-1b (pg/ml)
○BHS
•DS
Day Relative to Inoculation
-7 1 3 5 7 14 21 28

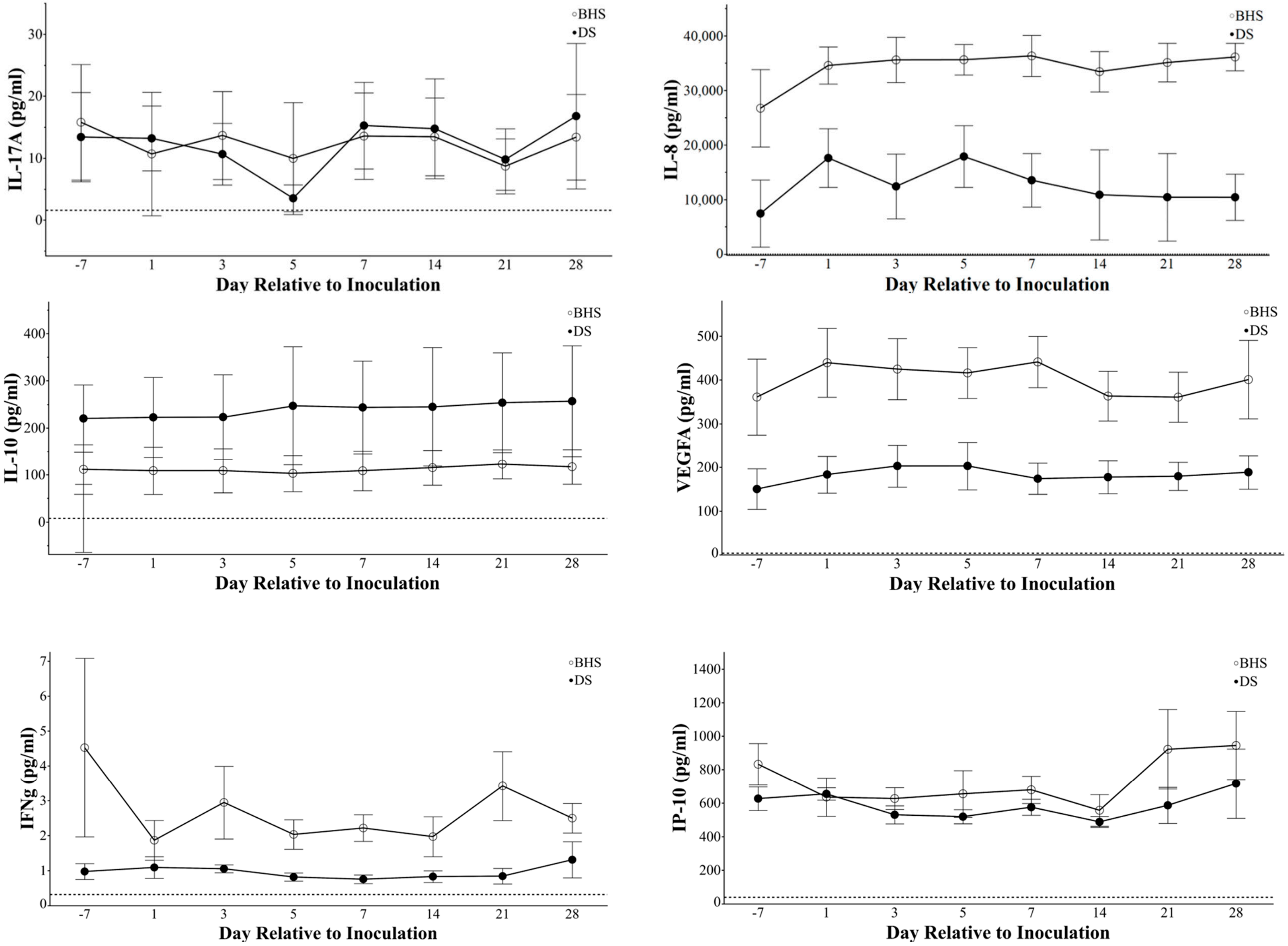

IL-17A (pg/ml)
30
20
10
0
-7
1
3
5
7
14
21
28
Day Relative to Inoculation
BHS
DS
IL-8 (pg/ml)
40,000
30,000
20,000
10,000
0
-7
1
3
5
7
14
21
28
Day Relative to Inoculation
BHS
DS
IL-10 (pg/ml)
400
300
200
100
0
-7
1
3
5
7
14
21
28
Day Relative to Inoculation
BHS
DS
VEGFA (pg/ml)
500
400
300
200
100
0
-7
1
3
5
7
14
21
28
Day Relative to Inoculation
BHS
DS
IFNg (pg/ml)
7
6
5
4
3
2
1
0
-7
1
3
5
7
14
21
28
Day Relative to Inoculation
BHS
DS
IP-10 (pg/ml)
1400
1200
1000
800
600
400
200
0
-7
1
3
5
7
14
21
28
Day Relative to Inoculation
BHS
DS

Supplemental Figure S6. Serum cytokines measured by multi-plex ELISA. The cytokine being measured is on the y-axis in pg/ml measurements while the day relative to inoculation is on the x-axis. Error bars represent the standard error. MIP-1a, MIP-1b, and IP-10 have secondary names as CCL3, CCL4, and CXCL10 respectively.
