# Supplementary material for: Differential Immunological Responses of Adult Domestic and Bighorn Sheep to Inoculation with Mycoplasma ovipneumoniae Type Strain Y98
Source: Microorganisms. 2024 Dec 21;12(12):2658. doi: 10.3390/microorganisms12122658 (PMC11728652; doi:10.3390/microorganisms12122658)
Supplement: Supplementary file 1 [file microorganisms-12-02658-s001.zip › Supplemental Figure S7 Antibody Time Course.pdf]

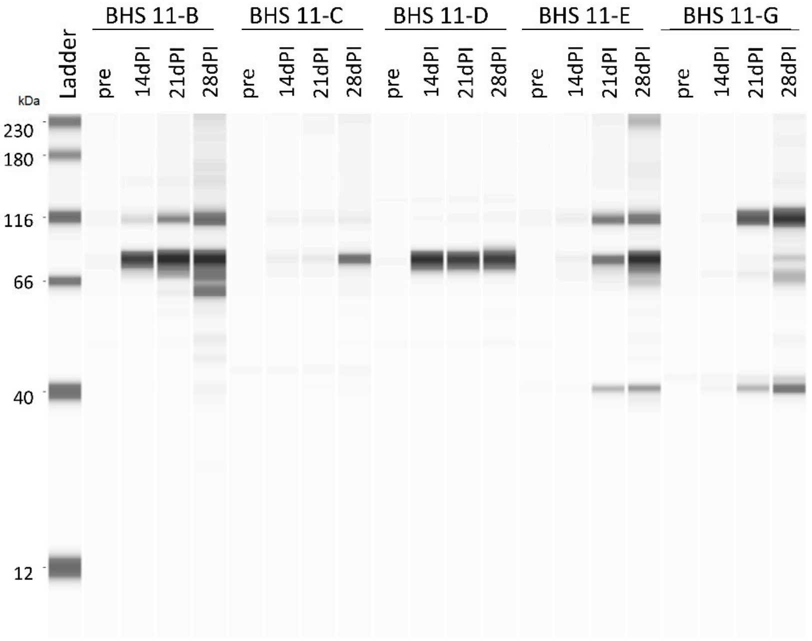

Supplemental Figure S7: Bighorn sheep serum antibody response to *Mycoplasma ovipneumoniae* over time. BHS serum collected pre-inoculation or 14-, 21-, and 28- days post inoculation (dPI) is used as primary antibody.
