# Supplementary material for: Differential Immunological Responses of Adult Domestic and Bighorn Sheep to Inoculation with Mycoplasma ovipneumoniae Type Strain Y98
Source: Microorganisms. 2024 Dec 21;12(12):2658. doi: 10.3390/microorganisms12122658 (PMC11728652; doi:10.3390/microorganisms12122658)
Supplement: Supplementary file 1 [file microorganisms-12-02658-s001.zip › Supplemental Table S1 Antibodies for Flow Cytometry.pdf]

**Supplemental Table S1: Flow cytometry antibodies and their recognized markers.**

| Primary Antibodies | | | | |
|---|---|---|---|---|
| **Leukocyte Differentiation Marker** | **Recognized Molecule, Reference** | **Antibody clone (Isotype)** | **Source** | **Staining Concentration** |
| CD11a | α integrin, [1] | HUH73A (IgG1) | WSU | 5μg/ml |
| CD11b | α integrin, [1] | MM12A (IgG1) | WSU | 5μg/ml |
| CD14 | CD14, [2] | CAM36A (IgG1) | WSU | 5μg/ml |
| CD16 | FcγRIIIA, [3] | VPM64 (IgG1) | Bio-Rad | 5μg/ml |
| CD18 | β2 integrin, [1] | BAQ30A (IgG1) | WSU | 5μg/ml |
| CD62L | L-selectin, [4] | DU1-29 (IgG1) | WSU | 5μg/ml |
| CD172a | Signal regulatory protein alpha (SIRP alpha), [5] | DH59B (IgG1) | WSU | 10μg/ml |
| **Secondary Antibodies** | | | | |
| **Secondary Isotype** | **Recognized Molecule, Reference** | **Alexa Fluor** | **Source** | **Staining Dilution** |
| goat anti-mouse $IgG_1$ | primary antibody | 647 | Southern Biotech #1070-31 | 1:1000 |

Primary and secondary antibodies utilized for flow cytometry. Primaries have their listed clonality and isotype, while the secondary has its listed fluorescence. The recognized surface molecule, source of purchase, and concentration/dilution are also given.


1. Mazzone, A. and G. Ricevuti, *Leukocyte CD11/CD18 integrins: biological and clinical relevance.* Haematologica, 1995. **80**(2): p. 161-75.
2. Haziot, A., B.Z. Tsuberi, and S.M. Goyert, *Neutrophil CD14: biochemical properties and role in the secretion of tumor necrosis factor-alpha in response to lipopolysaccharide.* J Immunol, 1993. **150**(12): p. 5556-65.
3. Yeap, W.H., et al., *CD16 is indispensable for antibody-dependent cellular cytotoxicity by human monocytes.* Scientific Reports, 2016. **6**.
4. Arbones, M.L., et al., *Lymphocyte homing and leukocyte rolling and migration are impaired in L-selectin-deficient mice.* Immunity, 1994. **1**(4): p. 247-60.
5. Konrad, F.M., et al., *How Adhesion Molecule Patterns Change While Neutrophils Traffic through the Lung during Inflammation.* Mediators of Inflammation, 2019. **2019**.
